# Establishment and validation of a novel risk model based on PANoptosis-related genes to predict prognosis in head and neck squamous cell carcinoma

**DOI:** 10.1097/MD.0000000000042299

**Published:** 2025-05-02

**Authors:** Yi-Fen Wu, Xiao-Hui Jiang, Dan-Ting Qian

**Affiliations:** a Department of Stomatology, People’s Hospital of Kecheng District, Quzhou City, Zhejiang, China.

**Keywords:** chemotherapy, HNSC, immune, PANoptosis, single-cell RNA

## Abstract

Head and neck squamous cell carcinoma (HNSC) is a common cancer worldwide with poor prognosis. Current treatment methods have limited effect on improving the prognosis of patients with HNSC. Differentially expressed PANoptosis-related genes in HNSC were identified from the TCGA using limma and WGCNA. A prognostic model was established using univariate and multivariate Cox regression analyses and machine learning, and its performance was evaluated using Kaplan–Meier and receiver operating characteristic curves. SNP data was analyzed using maftools package. Immune analysis was performed using IOBR package and TIDE website. The scRNA data was analyzed using Seurat and cellchat package. The expression of hub genes was validated in vitro. The prognostic model comprising 5 hub PANoptosis-related genes (AIFM1, AKT3, CDKN2A, EGFR, IL1RAP) accurately predicted patient outcomes, with the high-risk group exhibiting poorer survival. mRNA expression levels of all 5 hub genes were elevated in the tumor cells, but only AIFM1, AKT3 and IL1RAP’s protein expression were higher in tumor tissues. Additionally, high expression of AIFM1, AKT3, EGFR, IL1RAP and low expression of CDKN2A indicated poor prognosis of HNSC patients. The decreasing levels of CD4 T cells, CD8 T cells and M1 macrophages were observed in high-risk groups. There was a significant difference of 5-fluorouracil in low and high-risk groups. scRNA analysis exhibited that TNF pathway was important in the interaction between macrophages and T cells. We identified 5 hub genes and constructed a great risk model for the prognosis of HNSC. The immune cells may influence the HNSC malignant through TNF signal pathway.

## 1. Introduction

Head and neck squamous cell carcinoma (HNSC) is a highly prevalent and aggressive malignancy with poor prognosis, with over 900,000 new cases and 400,000 deaths worldwide every year.^[1,2]^ The common risk factors for HNSC include smoking, alcohol consumption, viral infections and genetic factors.^[3]^ It affects various sites including the oral cavity, pharynx, larynx and nasal cavity.^[4]^ Despite advancements in treatment modalities, the prognosis for HNSCC remains relatively poor,^[5]^ emphasizing the urgent need for improved diagnostic and therapeutic strategies. HNSC is characterized by complex molecular alterations, including genetic mutations, epigenetic modifications, and dysregulated signaling pathways.^[6,7]^ Genetic mutations were observed in almost 30% patients diagnosed with HNSC, and gene mutations could result in the dysregulation of key cellular processes, including cell proliferation, apoptosis and DNA repair.^[8,9]^ These alterations contribute to tumor initiation, progression, and therapy resistance.^[10]^ Therefore, understanding the underlying molecular mechanisms is crucial for the development of effective targeted therapies and personalized treatment approaches. Furthermore, dysregulated signaling pathways, including the PI3K/AKT/mTOR and the EGFR, are frequently observed in HNSCC.^[11,12]^ These pathways play crucial roles in cell growth, survival, and migration, and their dysregulation can promote tumor development and therapy resistance.^[13,14]^ Therefore, understanding the molecular mechanisms underlying HNSC can pave the way for the development of targeted therapies. Additionally, personalized treatment approaches, based on the individual molecular profile of each patient’s tumor, can be developed to optimize treatment outcomes.

In recent years, the identification and characterization of molecular biomarkers have emerged as promising tools for HNSC management.^[15–17]^ These biomarkers can serve as indicators of disease progression, prognosis, and treatment response. Additionally, they offer the potential for early detection and risk assessment, facilitating timely intervention and improved patient outcomes. PANoptosis represents a convergence of 3 key forms of cell death, including pyroptosis, apoptosis, and necroptosis.^[18]^ At present, PANoptosis has emerged as a significant area of interest in cancer research due to its potential impact on cancer development and progression.^[19,20]^ Dysregulation of PANoptosis usually disrupts the balance between cell proliferation and death, contributing to cancer initiation and evasion of cell death mechanisms.^[21]^ The inflammatory nature of PANoptosis, through pyroptosis, can influence the tumor microenvironment, either stimulating an immune response or creating a pro-tumorigenic environment.^[22]^ However, the function and prognosis value of PANoptosis in HNSC was not clear.

Therefore, in this study, we aimed to construct a risk model which could effectively predict the prognosis of HNSC and preliminary explore the potential mechanism.

## 2. Methods

### 2.1. Data collection

The mRNA expression, clinical data, and survival data (overall survival [OS] status and OS time) were acquired from the TCGA database. The raw microarray mRNA data underwent log2 transformation, with genes possessing multiple probes being averaged. Additionally, the single-cell RNA (scRNA) data (GSE195832) was retrieved from the GEO database. The PANoptosis genes were obtained from previous studies.^[23]^

### 2.2. The identification of differentially expressed genes

To identify differentially expressed genes (DEGs) between groups of interest, we employed the limma R package. First, we preprocessed the log2-transformed mRNA expression data to remove batch effects and normalize the expression values across samples. DEGs were defined based on FDR < 0.05 and |FoldChange| ≥ 1.5.

### 2.3. Functional enrichment analysis

Pathway enrichment analysis was performed on the identified DEGs to elucidate the biological processes and pathways associated with the observed gene expression changes. Gene ontology (GO) and kyoto encyclopedia of genes and genomes (KEGG) pathway analysis were conducted to highlight the functional annotations and pathways enriched in the DEG list. Additionally, the GSEA software was used for the GSEA analysis.

### 2.4. Weighted gene co-expression network analysis

To identify gene modules and explore their potential associations with clinical traits, we employed the weighted gene co-expression network analysis (WGCNA) package (Langfelder and Horvath, 2008). First, we filtered the mRNA expression data obtained from the TCGA database to remove low-expressed genes using a cutoff of < 1 transcripts per million. Next, we performed a log2 transformation of the remaining gene expression data. To construct a co-expression network, we calculated the pairwise correlation between genes using the biweight midcorrelation method. Then, we transformed the correlation matrix into an adjacency matrix by raising it to a soft-thresholding power, β, which was determined using the scale-free topology criterion. A scale-free network with a power value of β = 6 was selected for subsequent analysis.

Based on the adjacency matrix, we constructed a topological overlap matrix (TOM) to measure the network interconnectedness between genes. Next, we performed hierarchical clustering analysis using the TOM-based dissimilarity measure and the average linkage method to identify gene modules. The dynamic tree cutting algorithm was applied to identify modules with a minimum module size of 30 genes and a minimum height of 0.25. Each module was assigned a unique color for visualization.

Furthermore, we assessed the correlation between the gene modules and clinical traits of interest. We calculated the module eigengenes, representing the first principal component of each module, and correlated them with clinical traits using Pearson correlation analysis. Modules significantly associated with clinical traits were considered as potential biomarkers.

### 2.5. The construction of risk model

Univariate Cox analysis was first used to select the genes related to the prognosis of HNSC. Then, 3 machine learning algorithms, namely XGBoost, random forest, and LASSO Cox, were performed for the purpose of key gene selection. The analysis was performed using the R programming language, with the “xgboost,” “randomForest,” and “glmnet” packages utilized accordingly. Next, multivariate Cox analysis was used to determine the hub genes which has independent prediction values. Finally, the risk model was constructed by gene expression and regression coefficient as follows:


$$\begin{array}{l} RisksCore_{i}=\underset{i=1}{\overset{n}{\sum }}\;Exp_{i}*Coe_{i} \end{array}$$

### 2.6. Establishment of the nomogram

The “rms” package in R was utilized to construct a nomogram for the prognostic prediction model, incorporating Cox regression or random forest analysis. The nomogram visually represented the contribution of clinical variables and risk scores to survival predictions. Additionally, the “calibrate” package in R was used to generate calibration curves, assessing the accuracy of the model’s predictions by comparing observed and predicted survival probabilities.

### 2.7. Gene mutation analysis

The SNP data of HNSC patients was downloaded from the TCGA database, and the mutation analysis was performed using maftools R package.

### 2.8. Immune microenvironment analysis

The infiltration of immune cells of HNSC patients was calculated by the cibersort algorithm in the IOBR R package. Additionally, the tumor immune dysfunction and exclusion (TIDE, https://tide.dfci.harvard.edu/) was used to evaluate the potential clinical efficacy of immunotherapy in different risk groups and reflect the potential ability of tumor immune escape. A higher TIDE score is associated with poorer ICI efficacy.

### 2.9. Drug sensitivity

In this study, we employed the “oncoPredict” R package to perform drug sensitivity analysis and investigate the potential therapeutic response of cancer cells to different drugs. The gene expression profiles of cancer cells and corresponding drug response data were preprocessed, normalizing and removing technical variations. Drug sensitivity was predicted using a machine learning approach, trained on a reference dataset, to predict drug response based on gene expression profiles. The accuracy of the predictions was evaluated by comparing the predicted drug response values with actual drug response data. Candidate drugs with potential high therapeutic response were identified based on predefined cutoff values or statistical significance thresholds.

### 2.10. Quantitative real-time polymerase chain reaction (qRT-PCR)

At first, total RNA was first extracted using TRIzol® Reagent, and its concentration and purity were measured using a NanoDrop 2000 spectrophotometer, while the integrity of RNA was confirmed through 1% agarose gel electrophoresis stained with ethidium bromide. Subsequently, the extracted RNA was transcribed into cDNA using the SuperScript™ IV First-Strand Synthesis System in a 20 µL reaction mixture, incubating under specific conditions for reverse transcription. Following this, the synthesized cDNA was used as a template for real-time quantitative PCR in the Applied Biosystems QuantStudio 6 Flex Real-Time PCR System, employing PowerUp™ SYBR™ Green Master Mix for staining and amplification under thermal cycling conditions (30 seconds at 95°C, then 3 seconds at 95°C and 20 seconds at 60°C for 40 cycles). After the PCR reaction, the data was analyzed using dedicated software, with GAPDH serving as the internal reference gene. The relative expression levels of the target genes were calculated using the 2^-ΔΔCt^ method. The primer sequences are shown in Table 1.

**Table 1:** All primers in qRT-PCR experiments in this study.

Gene	Primer sequences (5’-3’)
AIFM1-F	TTACTATTCCTCCCAGCACCC
AIFM1-R	CACAATCCCCACGACCACTTT
AKT3-F	GCGATGTTACCATTGTGAAAG
AKT3-R	CTGAAAAGTTGTTGAGGGGAT
CDKN2A-F	GAGGGCTTCCTGGACACG
CDKN2A-R	TCTATGCGGGCATGGTTA
EGFR-F	GAGGACAGCATAGACGACA
EGFR-R	GAGGAGGTTGAGGAGCAG
IL1RAP-F	ATGCCTCAGAACGCTGCGATGA
IL1RAP-R	GTCCCGGTCCTGCCTAGTCCA
GAPDH-F	ACCCAGAAGACTGTGGATGG
GAPDH-R	TCAGCTCAGGGATGACCTTG

F = Forward, qRT-PCR = quantitative real-time polymerase chain reaction, R = Reverse.

### 2.11. Immunohistochemistry

Tumor tissues and adjacent non-tumor tissues were collected from patients diagnosed with HNSC at our hospital. All specimens were fixed in 10% formalin for 24 hours, embedded in paraffin, and sectioned into 4 μm thick slices. The sections were deparaffinized in xylene, rehydrated through a graded ethanol series, and subjected to antigen retrieval using citrate buffer for 15 minutes. After cooling, sections were rinsed with phosphate-buffered saline (PBS). Endogenous peroxidase activity was blocked by incubating with 3% hydrogen peroxide for 10 minutes, followed by a 30-minute incubation with 5% bovine serum albumin in PBS to reduce nonspecific binding. Primary antibodies for AIFM1, AKT3, and IL1RAP were applied overnight at 4°C. After washing, the sections were incubated with secondary antibodies (1:500, [supplier name]) for 1 hour at room temperature. The antigen-antibody complexes were visualized using diaminobenzidine (DAB) and counterstained with hematoxylin. The results were observed under a microscope.

### 2.12. Single-cell RNA (scRNA) analysis

In this study, we employed single-cell RNA sequencing (scRNA-seq) to analyze the transcriptional profiles of cells. The scRNA-seq data were processed using dimensionality reduction techniques, such as PCA or t-SNE, implemented in the “Seurat” or “Scikit-learn” R packages, to visualize and explore cellular heterogeneity. Clustering algorithms, including k-means or hierarchical clustering, were then applied to identify distinct cell populations. To annotate cell types, we used the SingleR algorithm and the “SingleR” R package, which compares the gene expression profiles of single cells against a reference dataset, such as the Human Cell Atlas, to assign putative cell types. We further investigated cell-cell communication using the “cellchat” R package, which integrated ligand-receptor interactions and gene expression data to infer potential cell communication networks. Statistical analysis, including differential expression analysis and enrichment analysis, was performed using appropriate tests and a significance level of *P* < .05. The methodology followed SCI writing standards, ensuring transparency and reproducibility.

### 2.13. Statistical analysis

In this study, the statistical operation was performed in SPSS 25 and R (version 4.3.1, Chicago). The difference between the 2 groups was compared with an independent sample t-test. The log-rank test was performed for the survival analysis. The Pearson test was performed for the correlation analysis. *P* < .05 was considered a significant difference.

## 3. Results

### 3.1. The identification and enrichment function analysis of DEG

**Figure 1.**
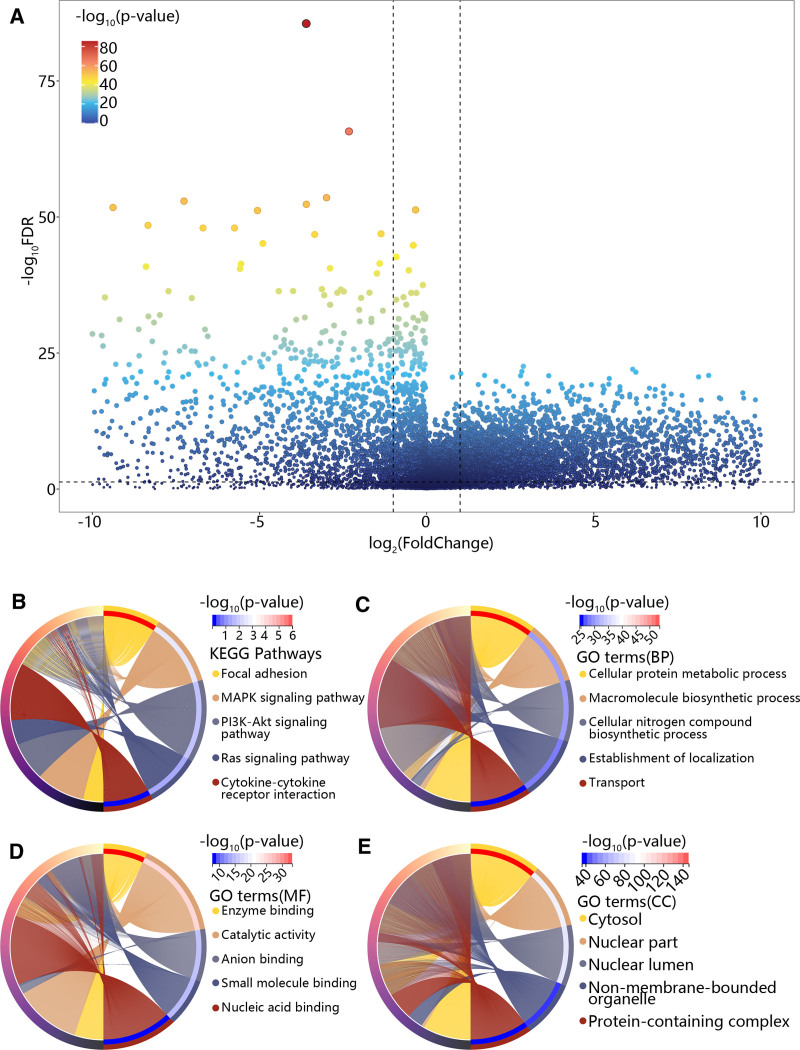
The identification and enrichment function analysis of DEG. (A) The volcano plot of DEGs. (B) The KEGG analysis of DEGs. (C) The GO analysis of DEGs in BP. (C) BP, (D) MF, and (E) CC enrichment analysis results of DEGs. BP = biological process, CC = cellular component, DEGs = differentially expressed genes, GO = gene ontology, KEGG = Kyoto encyclopedia of genes and genomes, MF = molecular function.

After the samples were divided into HNSC and control groups, the DEGs were identified using limma package. As shown in Figure 1A, 6147 upregulated genes and 2258 downregulated genes were obtained with |foldchanges| > 1.5. The KEGG analysis exhibited that these DEGs were enriched in focal adhesion, cytokine-cytokine receptor interaction, MAPK, PI3K-Akt and Ras signaling pathways (Fig. 1B). Besides, GO analysis showed that DEGs were mainly involved in the cellular protein metabolic process, macromolecule biosynthetic process and cellular nitrogen compound biosynthetic process in terms of biological process (Fig. 1C). The molecular function included enzyme binding, catalytic activity and anion binding (Fig. 1D). Furthermore, in the aspect of cellular component, DEGs mainly exist in the cytosol, nuclear part and nuclear lumen (Fig. 1E).

### 3.2. The identification of important modules using WGCNA

**Figure 2.**
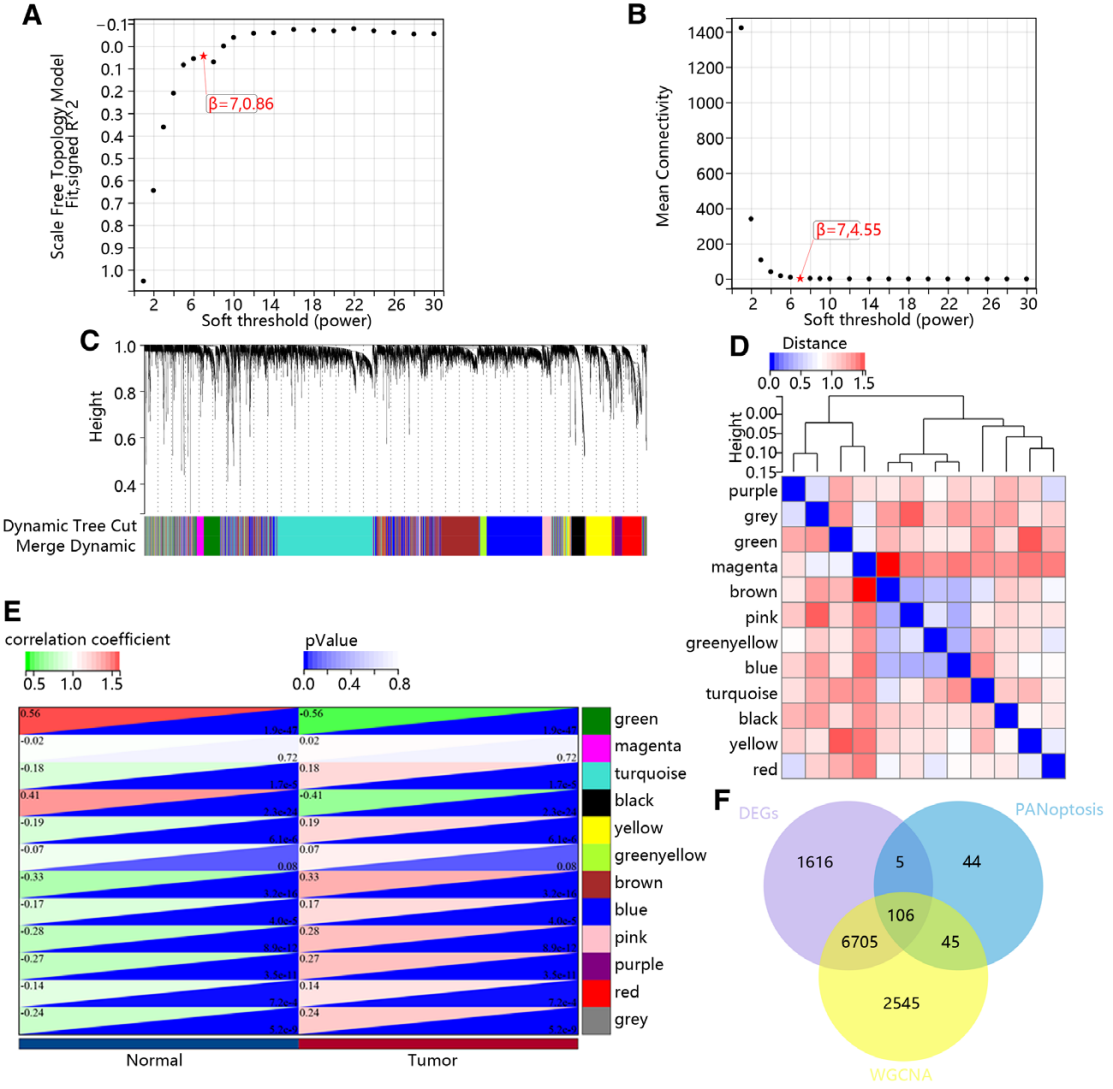
Identification of modules associated with the HNSC. (A and B) Analysis of network topology for various soft-thresholding powers. (C) Clustering dendrogram of genes, with dissimilarity based on topological overlap, together with assigned module colors. (D) Eigenvector gene clustering tree and module correlation thermographic. (E) Heatmap displaying the correlation between the co-expression modules and HNSC. (F) Venn diagram demonstrates overlapping genes of the DEG, PANoptosis and WGCNA. DEG = differentially expressed genes, HNSC = head and neck squamous cell carcinoma, WGCNA = weighted gene co-expression network analysis.

In this study, the power of β = 7 was selected as the soft-thresholding parameter to ensure a scale-free network (Fig. 2A and B). Then, 12 modules were identified (Fig. 2C), and the correlation among them was visualized in Figure 2D. Next, we calculated the correlation between modules and HNSC, the result identified that most modules were closely related to the pathogenesis of HNSC (Fig. 2E). Thus, the 106 overlapping genes were screened out based on modules genes, DEGs and PANoptosis-related genes (Fig. 2F).

### 3.3. The construction of risk model

**Figure 3.**
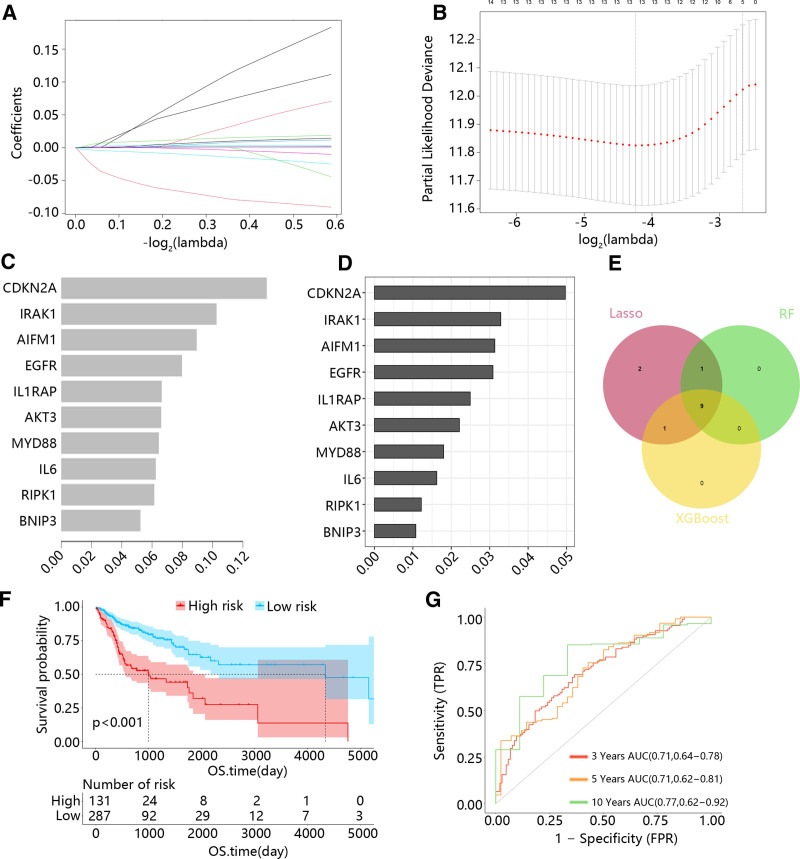
The risk model was constructed using Cox analysis and machine learning. (A and B) Ten-time cross-validation in the LASSO model and LASSO coefficient profiles. (C) The top 10 genes with the highest importance based on XGboost algorithms. (D) The top 10 genes with the highest importance based on RF algorithms. (E) Venn diagram demonstrates overlapping genes of the Laaso, RF, and XGBoost. (F) K–M curves for the OS of the high and low-risk group in the TCGA dataset. (G) Time-dependent ROC curves for 3-, 5-, and 10-yr survival in the TCGA dataset. ROC = receiver operating characteristic.

The univariate Cox analysis was first used to determine the prognosis-related genes, and 15 genes were screened out (Table S1, Supplemental Digital Content, https://links.lww.com/MD/O813). Then, the key genes from the 15 prognosis-related genes were identified using machine learning algorithms. In the LASSO regression analysis, an optimal lambda value was determined after 10 cross-validations, and 13 key genes were identified (Fig. 3A and B). According to the XGBoost and RF algorithms, the top 10 genes with the highest importance were selected = (Fig. 3C and D). Then, the 9 overlapping genes of 3 algorithms were considered as hub features (Fig. 3E). Next, 5 hub genes with independent predict values were determined using the multivariate Cox analysis (Table S2, Supplemental Digital Content, https://links.lww.com/MD/O814). According to the results of multivariate Cox analysis, the risk model was constructed, and the K–M curve showed that the high-risk group had a poor prognosis (Fig. 3F). Besides, the AUC value for HNSC at 3-, 5-, and 10-year was 0.71, 0.71, and 0.77, respectively (Fig. 3G).

### 3.4. The clinical characteristics of risk groups and the nomogram construction

**Figure 4.**
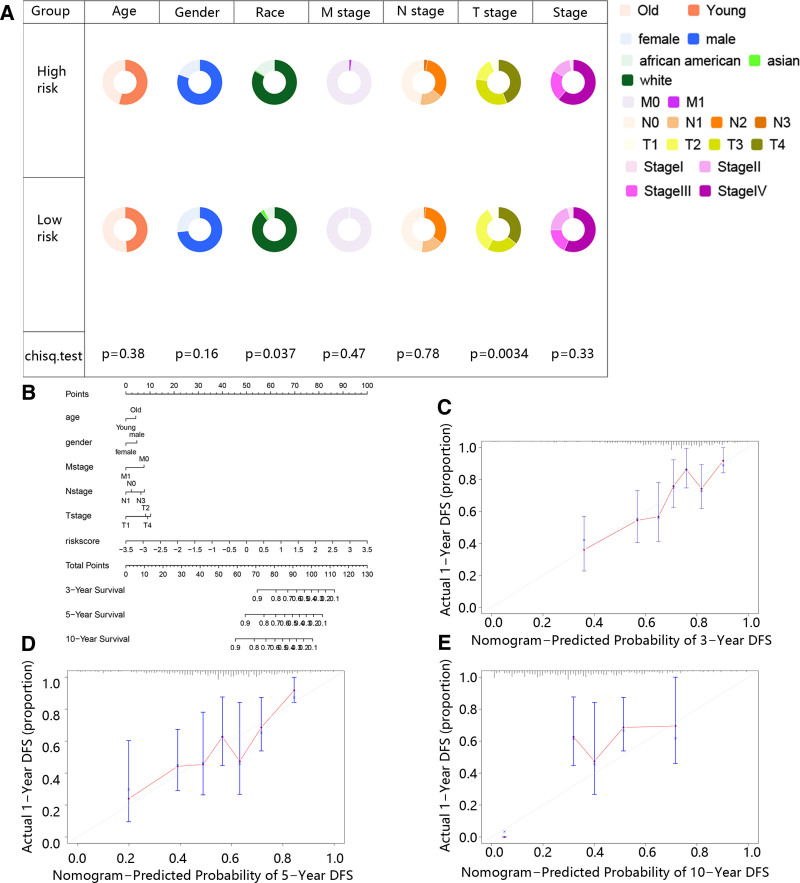
The establishment of nomograms based on the clinical characteristics and risk score. (A) The clinical characteristics in high and low-risk groups. (B) The establishment of nomogram. (C–E) The calibration curve of nomogram for the 3-, 5-, and 10-year survival rates.

Due to the prognosis of patients usually affected by clinical characteristics, we first assess the difference in clinical characteristics of patients in high and low-risk groups. The results exhibited that African-Americans had a higher proportion in the high-risk group, while White had a higher proportion in low-risk group (Fig. 4A). Besides, the patients in the T2 stage had an obviously higher proportion in low-risk group, while patients with T3 and T4 stage had an obvious higher proportion in high-risk group (Fig. 4A). In order to facilitate clinical research, a nomogram was established to predict 3-,5-,10-year survival rates based on clinical characteristics and risk score (Fig. 4B). Besides, the calibration curve demonstrated that the nomogram predicted the prognosis well (Fig. 4C and D).

### 3.5. Analysis of the somatic variants of high and low-risk group

**Figure 5.**
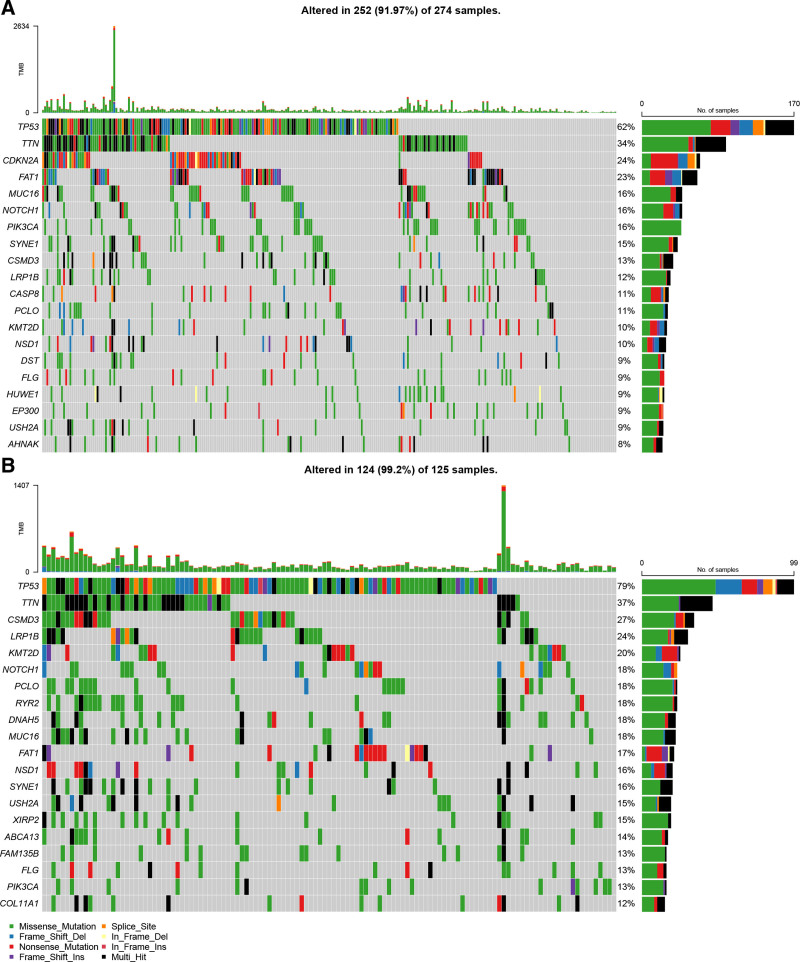
The mutation of genes in high and low-risk groups. (A) The mutation of genes in the low-risk group. (B) The mutation of genes in the high-risk group.

The mutation of genes was closely related to the development of tumors. Therefore, we analyzed the differences in somatic mutations between high and low-risk groups in the TCGA dataset. As shown in Figure 5A and B, the primary genetic mutations in the 2 risk groups were both TP53 and TTN. Besides, we found that the mutation of CDKN2A had a high mutation proportion (24%) in the low-risk group, while it had a low mutation proportion in the high-risk group (<12%).

### 3.6. The immune microenvironment and immunotherapy response

**Figure 6.**
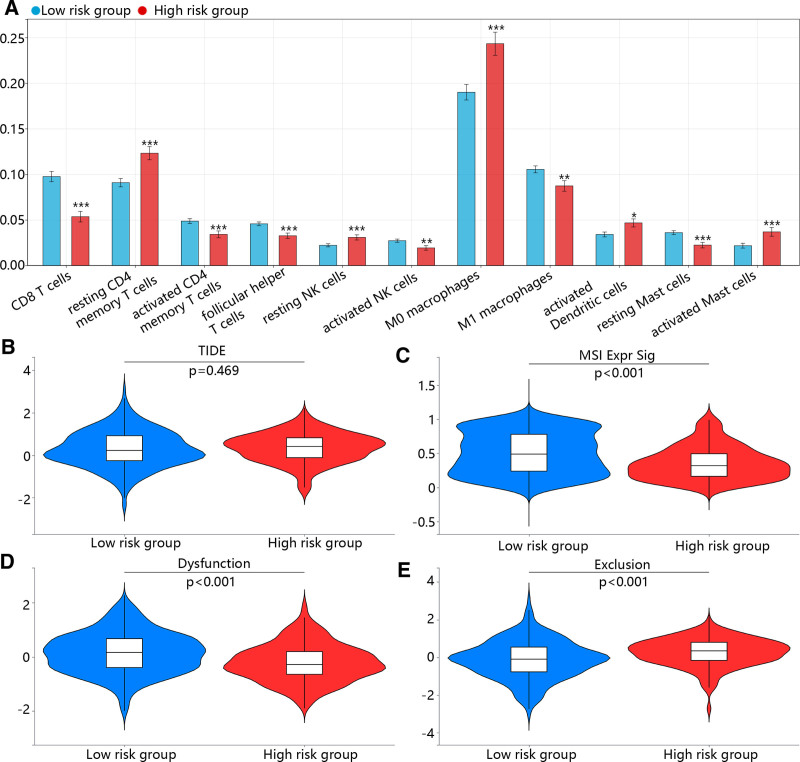
The immune infiltration analysis and immunotherapy prediction. (A) The immune infiltration levels were calculated by the cibersort algorithm. (B–E) The (B) TIDE, (C) MSI, (D) dysfunction and (E) exclusion scores in low and high-risk groups. TIDE = tumor immune dysfunction and exclusion, MSI = microsatellite instability.

The immune microenvironment also plays an important role in the treatment and prognosis of patients with tumors. As shown in Figure 6A, the immune infiltration levels of CD8 T cells, activated CD4 T cells, follicular helper T cells, activated NK cells and M1 macrophages were significantly downregulated, while M0 macrophages and dendritic cells and activated mast cells were all significantly upregulated in high-risk group compared with low-risk group. Then, we assessed the immunotherapy response using TIDE. The results indicated that the TIDE score had no significant difference between low and high-risk groups (Fig. 6B). Besides, microsatellite instability and dysfunction scores were significantly higher in low-risk group compared with the high-risk group (Fig. 6C and D), while exclusion score was lower in low-risk group (Fig. 6E).

### 3.7. Risk score guided chemotherapy strategies

**Figure 7.**
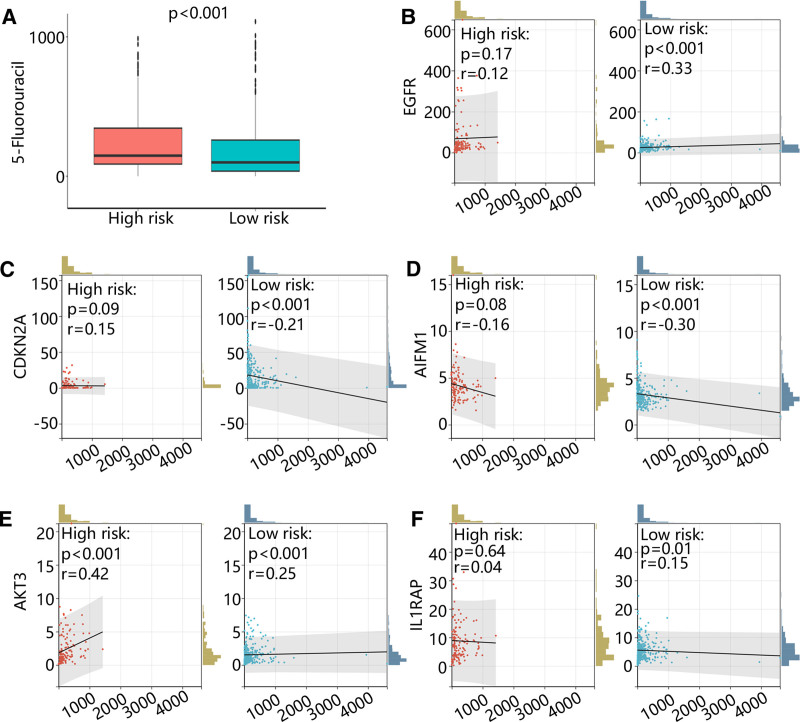
The drug sensitivity analyzed by oncoPredict. (A) The drug sensitivity of 5-fluorouracil in high and low-risk groups. (B–F) The correlation between sensitivity of 5-fluorouracil and (B) EGFR expression, (C) CDKN2A expression, (D) AIFM1 expression, (E) AKT3 expression, (F) IL1RAP expression.

Except for immunotherapy, chemotherapy is also vital for the prognosis of patients with malignant tumors. The results of the drug sensitivity analysis showed that the high-risk group was more sensitive to 5-fluorouracil (Fig. 7A). Next, to explore the function of hub genes in the chemotherapy, we calculated the correlation between hub gene expressions and drug sensitivity of 5-fluorouracil. It was obvious that 5 hub genes were all closely related to the sensitivity of 5-fluorouracil in low-risk group, but AKT3 was strong and positively related to the sensitivity of 5-fluorouracil in the high-risk group (Fig. 7B–F).

### 3.8. The expression of hub genes and survival analysis

**Figure 8.**
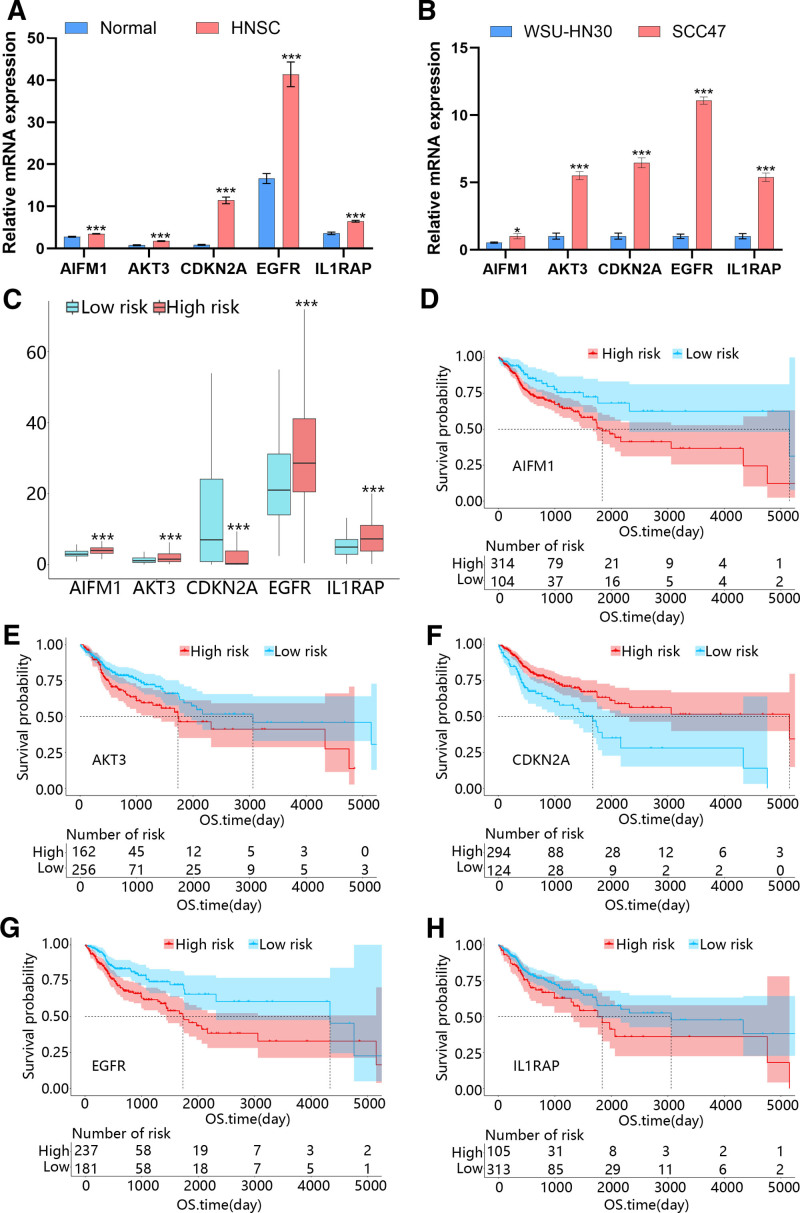
The expression and predictive value of hub genes in HNSC. (A) The expression of hub genes in normal and HNSC samples. (B) The expression in WSU-HN30 and SCC47 cells. (C) The expression of hub genes in low and high-risk groups. (D-H) The K–M curve of the HNSC patients in low and high expression of (D) AIFM1, (E) AKT3, (F) CDKN2A, (G) EGFR, (H) ILRAP. HNSC = head and neck squamous cell carcinoma.

Then, we analyzed the expression difference of hub genes in different groups. As shown in Figure 8A, the expression of 5 hub genes was all upregulated in the HNSC group compared with the normal group, which was consistent with our qRT-PCR results (Fig. 8B). Besides, from Figure 8C, it can be seen that AIFM1, AKT3, EGFR and IL1RAP were highly expressed in high-risk group, while CDKN2A expression was downregulated. Besides, the K–M curve indicated that the high expression of AIFM1, AKT3, EGFR and IL1RAP was related to the poor prognosis, but poor expression of CDKN2A was related to poor prognosis of HNSC patients (Fig. 8D–H).

**Figure 9.**
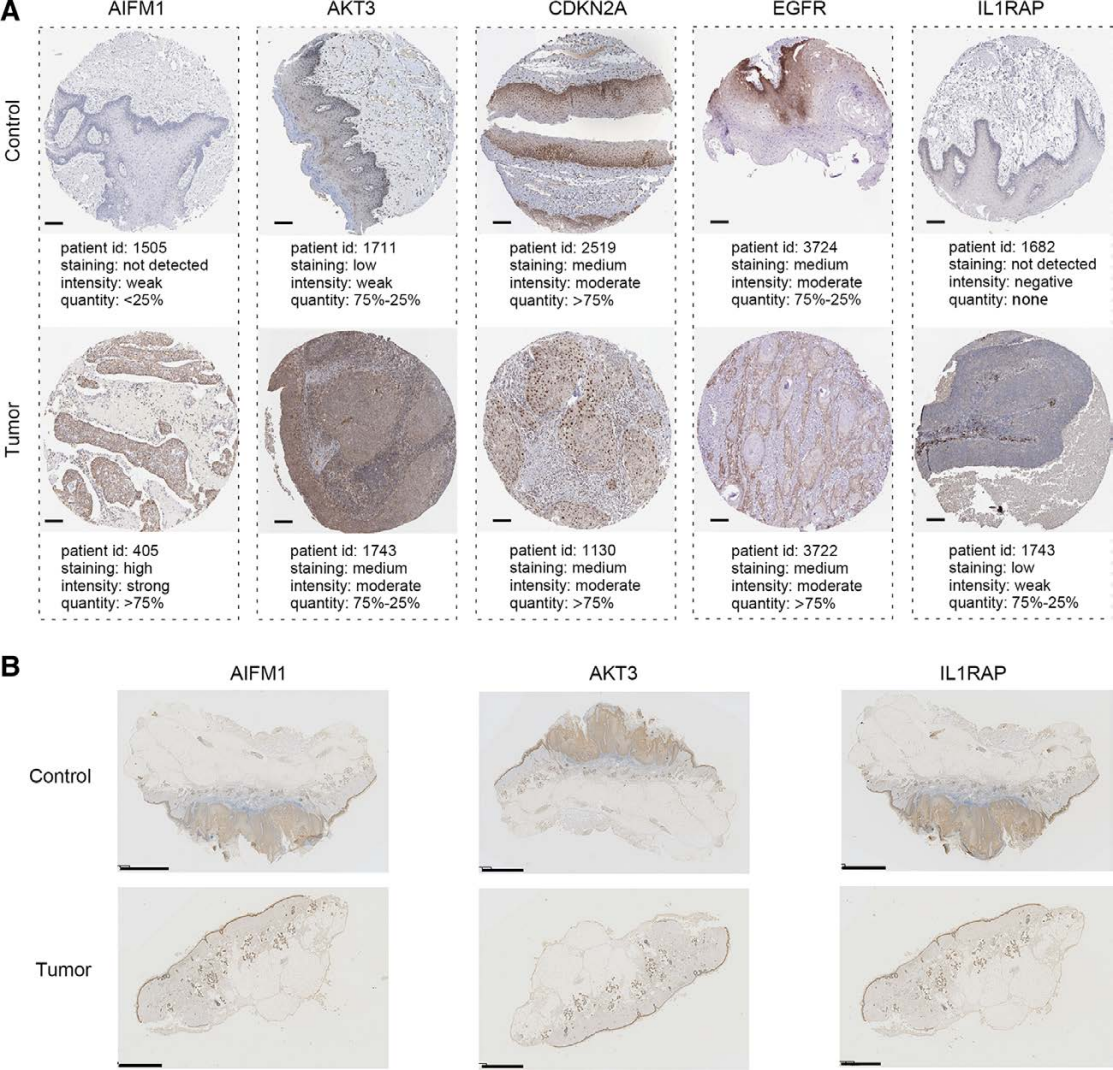
Protein expression of hub genes in HNSC. (A) The protein expression data of hub genes from the HPA database; scale bar = 100 μm. (B) Immunohistochemistry revealed the protein expression of AIFM1, AKT3 and IL1RAP; scale bar = 2.5 mm. HNSC = head and neck squamous cell carcinoma, HPA = human protein atlas.

Furthermore, we obtained immunohistochemical images of 5 hub genes in HNSC from The Human Protein Atlas (HPA) database. As shown in Figure 9A, compared to the control group, the tumor group exhibited higher positive expression of AIFM1, AKT3, and IL1RAP. There was no significant difference in the expression levels of CDKN2A and EGFR between the 2 groups. The AIFM1, AKT3, and IL1RAP were then selected for experimental validation, and the results were consistent with the data from HPA (Fig. 9B).

### 3.9. The function analysis of hub genes

**Figure 10.**
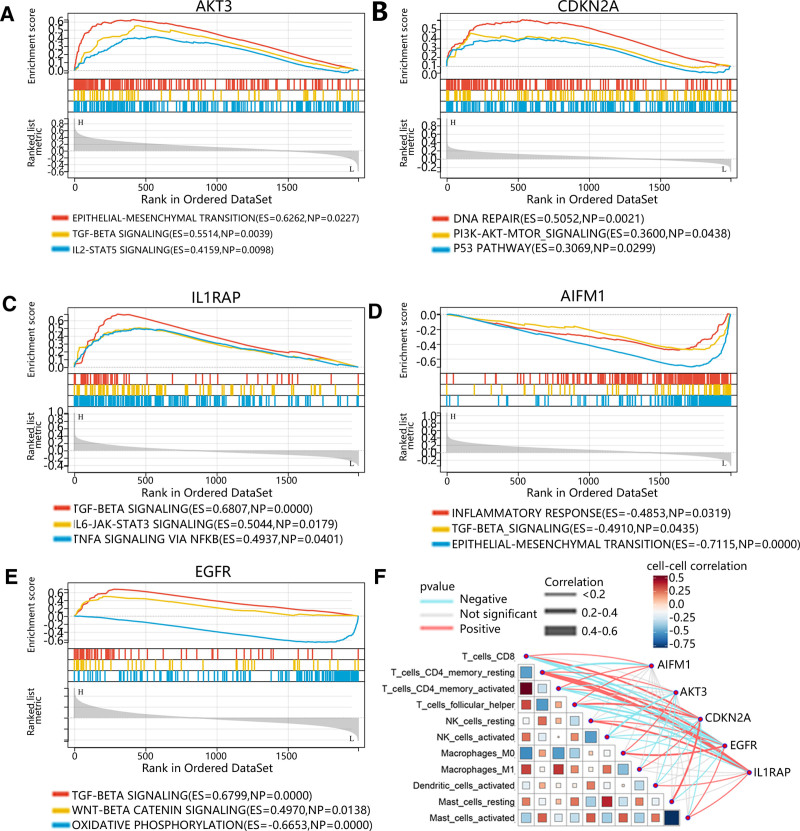
The GESA and correlation analysis of hub genes. (A–E) The GSEA analysis of (A) AKT3, (B) CDKN2A, (C) IL1RAP, (D) AIFM1 and (E) EGFR. (F) The correlation between hub genes and immune cells.

Then, in order to further the function of hub genes in the development of HNSC, the GSEA analysis was performed. The results demonstrated that high expression of AKT3 was enriched in epithelial-mesenchymal transition, TGF-beta signaling and IL2-STAT5 signaling (Fig. 10A). In addition, the high expression of CDKN2A was mainly involved in the DNA repair, PI3K-Akt-MTOR signaling and P53 pathway (Fig. 10B). Furthermore, the high expression of IL1RAP was enriched in TGF-beta signaling, IL6-JAK-STAT3 signaling and TNF-α signaling via NF-κB (Fig. 10C). Additionally, low expression of AIFM1 was associated to the inflammatory response, TGF-beta signaling and epithelial-mesenchymal transition (Fig. 10D). Besides, the high expression of EGFR was enriched in TGF-beta signaling AND WNT beta catenin signaling, and the low expression of EGFR was enriched in oxidative phosphorylation (Fig. 10E). The above results indicated that hub genes may be related to the immune infiltration. Therefore, we calculated the correlation between hub genes and immune cells, and the results were visualized in Figure 10F.

### 3.10. The scRNA analysis

**Figure 11.**
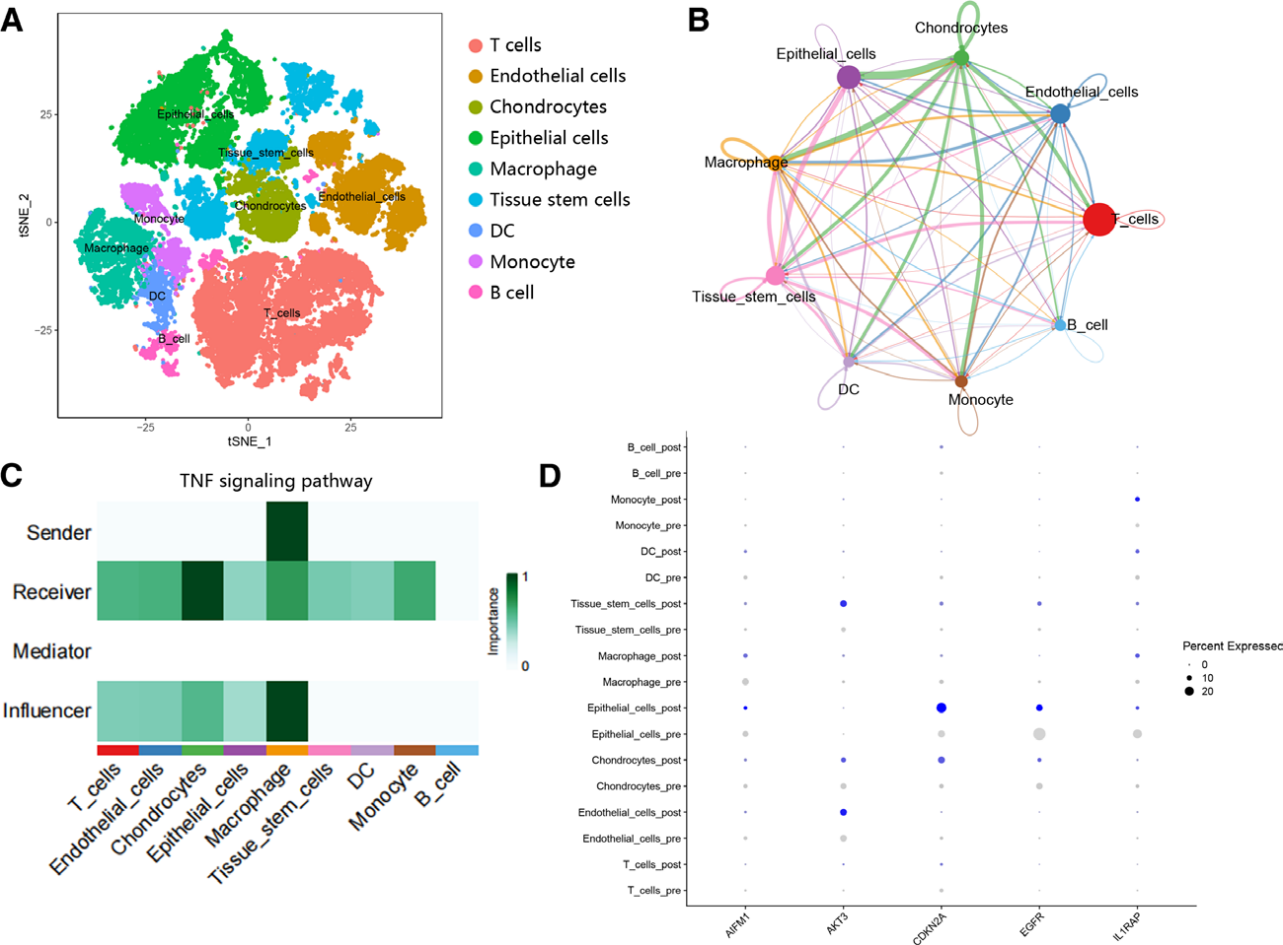
The verification of hub genes through scRNA data. (A) The t-SNE plots of cell types. (B) The interaction among 9 cell types. (C) The cell interaction in the TNF signaling pathway. (D) The expression of hub genes in 9 cell types before and after immunotherapy.

The scRNA data was used to further analyze the potential mechanism of hub genes and immune response. From 8 samples, we obtained 56,582 cells at first, and 37,544 cells were used for the analysis after filter with the screening criteria. After the cells were screened out, the top 15 PCs were used for t-SNE, and 9 cell types were obtained, including T cells, endothelial cells, chondrocytes, epithelial cells, macrophages, tissue stem cells, DC, monocyte and B cells (Fig. 11A). The results indicated that T cell may play an essential role in the cell interaction (Fig. 11B), and T cells may be affected by macrophage by TNF signaling pathway (Fig. 11C). Besides, according to Figure 10D, it can be seen that hub genes were mainly expressed in epithelial cells, and the expression was changed after immunotherapy (Fig. 11D).

## 4. Discussion

Given the escalating incidence of head and neck squamous cell carcinoma (HNSC) on a global scale, there exists a demand for reliable molecular biomarkers due to the heterogeneous nature of this disease.^[24]^ Ferroptosis, an emerging form of programmed cell death, plays a multifaceted role in tumor development.^[25]^ However, research on ferroptosis in the context of HNSC remains limited.^[26]^

The prognosis of HNSC is heavily reliant on the disease stage at the time of diagnosis. Early-stage tumors are more amenable to treatment and exhibit a more favorable prognosis compared to advanced-stage tumors.^[27]^ In this study, we identified 5 pivotal genes (AIFM1, AKT3, CDKN2A, EGFR, IL1RAP) that can independently predict the prognosis of HNSC and constructed a robust risk model. The Kaplan–Meier and receiver operating characteristic curves demonstrated the excellent prognostic value of our risk model.

AIFM1 is a protein located on chromosome Xq26.1 and plays a vital role in apoptosis.^[28]^ Several studies have shown that AIFM1 can induce apoptosis to impede tumor development.^[29–31]^ However, our results revealed that AIFM1 acts as an oncogene and is highly expressed in HNSC. This implies that the function of AIFM1 may be contingent upon the specific tissue and cell type. Furthermore, AKT3 is a subtype of AKT.^[32]^ Divergent functions of AKT3 have been observed in breast cancer (BRCA) and lung adenocarcinoma (LUAD).^[33]^ Our findings demonstrated that AKT3 is highly expressed and promotes the progression of HNSC. Additionally, EGFR is a receptor tyrosine kinase within the ErbB family.^[34]^ Consistent with previous studies,^[35]^ we observed high expression of EGFR in HNSC, which is closely associated with poor prognosis. Moreover, IL1RAP is overexpressed in various tumors, including pancreatic cancer and triple-negative breast cancer,^[36,37]^ and elevated IL1RAP promotes tumor development through multiple oncogenic signaling pathways.^[38,39]^ Similarly, we discovered that IL1RAP is upregulated in HNSC and strongly correlated with poor prognosis. CDKN2A, also known as p16, is one of the most commonly altered genes in human cancers.^[40]^ It has been established as a tumor suppressor in multiple malignancies, including glioblastoma (GBM) and colorectal cancer (CRC).^[41,42]^ Furthermore, Zheng et al claimed that patients with CDKN2A mutations have a higher risk of developing HNSC.^[43]^ Further studies have shown that CDKN2A mutations often result in increased cell proliferation.^[44,45]^ Conversely, Lim et al demonstrated that CDKN2A disruption is not significantly associated with poor prognosis in oral tongue squamous cell carcinomas.^[46]^ Our results revealed that CDKN2A acts as an oncogene and is upregulated in the high-risk group, while CDKN2A mutations are more prevalent in the low-risk group. We hypothesize that CDKN2A mutations may play a crucial role in tumorigenesis by regulating the cell cycle of tumors and highly expressed CDKN2A may contribute to the malignancy of HNSC by mediating certain cancer-promoting signaling pathways.

The tumor microenvironment (TME) in HNSC often exhibits a highly immunosuppressive nature.^[47]^ Within the immune system, CD8 T cells play a pivotal role,^[48]^ and their positive function in antitumor immunity has been extensively documented, including in HNSC.^[4]^ Moreover, recent research has highlighted the importance of memory T cells in the development of HNSC.^[49]^ Studies have also shown that the activated TNF signaling pathway can inhibit T cell proliferation.^[50]^ On 1 hand, M1 macrophages can produce pro-inflammatory factors like TNF-α.^[51]^ On the other hand, TNF can induce the polarization of M1 macrophages by activating the NF-κB pathway.^[52]^ Han et al found a higher abundance of M1 macrophages and CD8 T cells in a low-risk HNSC population based on inflammatory response-related characteristics.^[53]^ Our results indicated a decreased infiltration of M1 macrophages in the high-risk group, and single-cell RNA sequencing results illustrated macrophage-T cell interactions mediated by the TNF signaling pathway. These findings suggest that downregulated M1 macrophages may promote T cell infiltration by inhibiting the TNF signaling pathway. However, we also observed a decrease in CD8 T cells and memory CD4 T cells in the high-risk group, and we found that the highly expressed AIFM1, AKT3, EGFR, and IL1RAP were enriched in the TGF-beta signaling pathway. A previous study revealed that AKT3 knockdown could attenuate the TGF-beta signaling.^[54]^ Simultaneous inhibition of EGFR and TNF signaling suppresses HNSC tumor growth.^[55]^ Additionally, the TGF-beta signaling pathway inhibits T cell infiltration.^[56,57]^ Therefore, we infer that hub genes may downregulate the infiltration of CD4 and CD8 T cells, thereby promoting the development of HNSC through the activation of the TGF-beta signaling pathway.

In conclusion, we identified 5 pivotal genes and constructed a robust risk model with excellent prognostic value. Furthermore, we found that TGF-beta may play a crucial role in immune responses and the malignancy of HNSC. Nevertheless, there are certain limitations to consider. Firstly, the data utilized in this study was solely obtained from the TCGA database and should be validated in other datasets with a larger sample size. Secondly, the findings from bioinformatics analysis should be further validated through in vivo and in vitro experiments.

## Author contributions

**Conceptualization:** Yi-Fen Wu.

**Formal analysis:** Yi-Fen Wu, Dan-Ting Qian.

**Funding acquisition:** Yi-Fen Wu.

**Investigation:** Xiao-Hui Jiang.

**Methodology:** Xiao-Hui Jiang, Dan-Ting Qian.

**Writing – original draft:** Yi-Fen Wu, Xiao-Hui Jiang, Dan-Ting Qian.

**Writing – review & editing:** Yi-Fen Wu.

## Supplementary Material


