# Supplementary material for: Establishment and validation of a novel risk model based on PANoptosis-related genes to predict prognosis in head and neck squamous cell carcinoma
Source: Medicine (Baltimore). 2025 May 2;104(18):e42299. doi: 10.1097/MD.0000000000042299 (PMC12055095; doi:10.1097/MD.0000000000042299)
Supplement: Supplementary file 1 [file medi-104-e42299-s001.docx]

**Table S1** The results of univariate Cox analysis

| Gene | HR | Lower | Upper | pvalue |
| --- | --- | --- | --- | --- |
| EGFR | 1.003 | 1.001 | 1.005 | <0.001 |
| CDKN2A | 0.975 | 0.960 | 0.990 | 0.002 |
| IRAK1 | 1.010 | 1.004 | 1.016 | 0.002 |
| RIPK1 | 0.830 | 0.736 | 0.937 | 0.003 |
| IL1RAP | 1.039 | 1.013 | 1.066 | 0.003 |
| PIK3CA | 1.166 | 1.047 | 1.299 | 0.005 |
| BNIP3 | 1.028 | 1.008 | 1.048 | 0.007 |
| IL6 | 1.009 | 1.002 | 1.016 | 0.007 |
| FADD | 1.028 | 1.007 | 1.050 | 0.009 |
| AIFM1 | 1.140 | 1.026 | 1.267 | 0.016 |
| MYD88 | 0.974 | 0.952 | 0.997 | 0.025 |
| GZMB | 0.981 | 0.965 | 0.999 | 0.035 |
| AKT3 | 1.092 | 1.006 | 1.186 | 0.038 |
| BMF | 0.898 | 0.811 | 0.995 | 0.040 |
| AKT1 | 1.044 | 1.001 | 1.088 | 0.045 |
