# Supplementary material for: Establishment and validation of a novel risk model based on PANoptosis-related genes to predict prognosis in head and neck squamous cell carcinoma
Source: Medicine (Baltimore). 2025 May 2;104(18):e42299. doi: 10.1097/MD.0000000000042299 (PMC12055095; doi:10.1097/MD.0000000000042299)
Supplement: Supplementary file 2 [file medi-104-e42299-s002.docx]

**Table S2** The results of multivariate Cox analysis

| Gene | HR | Lower | Upper | pvalue |
| --- | --- | --- | --- | --- |
| AIFM1 | 1.252 | 1.131 | 1.385 | <0.001 |
| AKT3 | 1.094 | 1.015 | 1.179 | 0.018 |
| BNIP3 | 1.022 | 1 | 1.044 | 0.054 |
| CDKN2A | 0.972 | 0.956 | 0.988 | 0.001 |
| EGFR | 1.003 | 1.001 | 1.005 | 0.004 |
| IL1RAP | 1.035 | 1.007 | 1.064 | 0.014 |
